# Renal Fibrosis in Lupus Nephritis

**DOI:** 10.3390/ijms232214317

**Published:** 2022-11-18

**Authors:** Savino Sciascia, Martina Cozzi, Alice Barinotti, Massimo Radin, Irene Cecchi, Roberta Fenoglio, Daniele Mancardi, Georgia Wilson Jones, Daniela Rossi, Dario Roccatello

**Affiliations:** University Center of Excellence on Nephrologic, Rheumatologic and Rare Diseases (ERK-net, ERN-Reconnect and RITA-ERN Member) with Nephrology and Dialysis Unit, Center of Immuno-Rheumatology and Rare Diseases (CMID), Coordinating Center of the Interregional Network for Rare Diseases of Piedmont and Aosta Valley (North-West Italy), Department of Clinical and Biological Sciences, San Giovanni Bosco Hub Hospital, University of Turin, 10154 Turin, Italy

**Keywords:** renal fibrosis, lupus nephritis, interstitial fibrosis, tubular atrophy, systemic lupus erythematosus

## Abstract

Fibrosis can be defined as a pathological process in which deposition of connective tissue replaces normal parenchyma. The kidney, like any organ or tissue, can be impacted by this maladaptive reaction, resulting in persistent inflammation or long-lasting injury. While glomerular injury has traditionally been regarded as the primary focus for classification and prognosis of lupus nephritis (LN), increasing attention has been placed on interstitial fibrosis and tubular atrophy as markers of injury severity, predictors of therapeutic response, and prognostic factors of renal outcome in recent years. This review will discuss the fibrogenesis in LN and known mechanisms of renal fibrosis. The importance of the chronicity index, which was recently added to the histological categorization of LN, and its role in predicting treatment response and renal prognosis for patients with LN, will be explored. A better understanding of cellular and molecular pathways involved in fibrosis in LN could enable the identification of individuals at higher risk of progression to chronic kidney disease and end-stage renal disease, and the development of new therapeutic strategies for lupus patients.

## 1. Introduction

Fibrosis can be defined as a pathological process in which deposition of connective tissue replaces normal parenchyma. It represents a maladaptive response to prolonged injury or chronic inflammation, and any organ or tissue can be affected. In normal conditions, following an acute insult, several cellular and molecular players are activated and participate to what is known as “wound healing” process. This process is auto-limited and represents a fundamental mechanism of tissue repair and regeneration. However, if injury becomes a repeated or prolonged phenomenon, normal healing shifts to fibrogenesis and scarring. The common mechanism underlying fibrogenesis relies on the sustained production of cytokines, angiogenic factors, and growth factors that lead to an imbalance between extracellular matrix (ECM) production by myofibroblasts and degradation by matrix metalloproteinases (MMPs) [1]. This imbalance leads to excessive ECM deposition and consequent substitution of normal parenchyma with non-functional connective tissue [2]. Progressive architectural changes and scarring of involved tissues lead to irreversible organ dysfunction and eventual failure [3]. Some authors even suggested that fibrosis can be seen as a persistent autoimmune response involving concomitant inflammation, remodeling, and altered healing processes [4].

Injuries may affect in first place glomerular tufts, as in glomerulonephritides, or may affect the tubular compartment with different mechanisms, as direct tubular toxicity or obstructive uropathy. Progressive accumulation of ECM and loss of functional parenchyma lead to impairment of renal functions, chronic kidney disease (CKD), and, eventually, end stage renal disease (ESRD) [5]. It is well known that interstitial fibrosis and tubular atrophy (IF/TA), regardless of the initial trigger, strongly correlate with kidney function and renal outcomes [6,7,8].

Renal fibrosis has been detected and studied in several kidney diseases, including lupus nephritis (LN). LN affects approximately 40% of adults and 80% of children suffering from systemic lupus erythematosus (SLE) [9]. Even after considerable advances in therapeutic strategies and improvements in survival rates for SLE patients, LN remains a leading cause of morbidity and mortality [10], with 10% of patients reaching ESRD and consequent need of renal replacement therapy, either dialysis or transplantation. While glomerular injury has long been considered as the main focus for classification and prognosis of LN, in recent years more importance has been progressively attributed to IF/TA as a marker of severity of injury, prediction of response to therapy, and prognosis of renal outcome.

In this review, we will address known mechanisms of renal fibrosis and we will focus on fibrogenesis in LN. We will discuss recent changes in histological classification of LN, with the introduction of chronicity index [11] and its role in prediction of response to therapy and renal prognosis for patients affected by LN. Finally, we will discuss possible future therapies targeting renal fibrosis.

## 2. Renal Fibrogenesis

Renal fibrogenesis is a complex maladaptive process that can be initiated by a variety of injurious insults, either targeting the glomeruli or the tubular compartment. Fibrosis represents a final common route that contributes to progressive loss of kidney function, development of CKD, and, eventually, ESRD.

In all tissues, including the kidneys, fibrogenesis shares similar mechanisms. It is well accepted that, after an acute insult, resident mesenchymal cells, fibroblasts, and pericytes, are activated and start a pro-inflammatory cascade aimed at repairing the injury, with secretion of cytokines, chemokines, reactive oxygen species (ROS), and recruitment of leukocytes [3,4,6]. This pro-inflammatory environment triggers a proliferation of myofibroblasts, which in turn secrete collagen and promote deposition of ECM. In normal conditions, repair of injured tissue is self-limited, and the triggered cascade ends with apoptosis of activated myofibroblasts. If the inflammatory stimulus continues to be present or a repeated insult persists, the whole repair process becomes unbalanced, leading to excessive ECM deposition and fibrogenesis, with distortion of parenchymal architecture and progressive functional impairment [6]. In the kidney, fibrosis leads to scarring of glomerular tufts and tubular compartments, with loss of working nephrons and reduction of renal function [7].

Many different cellular and molecular pathways have been identified as key players in the development of renal fibrosis, and a detailed discussion of all the studied mechanisms is beyond the scope of this review. We will briefly touch on the main players involved in renal fibrogenesis, including myofibroblasts, epithelial-mesenchymal transition (EMT), macrophages, transforming growth factor- β (TGF-β) signaling, and hypoxia.

### 2.1. Myofibroblasts and EMT

Myofibroblasts are generally considered as the predominant effectors of fibrogenesis, in kidneys as well as in other organs, such as lungs [6,12]. They have features of both fibroblasts and smooth muscle cells, co-expressing α-smooth muscle actin (αSMA) and other markers including collagens, vimentin, platelet-derived growth factor receptor-β (PDGFR-β), fibroblast-specific protein 1 (FSP-1), and CD73 [13]. They produce collagen contributing to ECM deposition and repair of injured tissues, undergoing apoptosis when the healing process is completed. However, if the injurious triggers persist, the process is not stopped and myofibroblasts are stimulated to continue the production of collagen and ECM deposition. The origin of these cells in renal fibrosis has long been debated and still represents a controversial issue. It has been suggested that myofibroblasts in renal fibrotic tissue may derive from different precursor resident cells, including fibroblasts, pericytes, tubular epithelial cells, and endothelial cells [14]. While their precise origin has not been fully defined yet, it is well-established that they represent key players in renal fibrogenesis, as it is in other tissues. Myofibroblasts not only contribute to deposition of ECM, but they can directly induce generation of radical oxygen species, affect cell proliferation via PDGFs, and alter renal tissue architecture through their intrinsic contractile properties [14]. Their pathogenic role in renal fibrosis has been demonstrated in different murine models in which the ablation of myofibroblasts can attenuate progression of renal fibrosis [13,15]. Myofibroblasts represent also the ending point of a major process involved in fibrogenesis, namely epithelial to mesenchymal transition (EMT). EMT represents a multi-step phenotypic conversion from epithelial to mesenchymal phenotype, in which tubular epithelial cells (TECs) sequentially start to express mesenchymal markers, such as α-SMA, and undergo spatial reorganization, with loss of adhesion to tubular basal membrane and migration to the interstitial compartment as mesenchymal cells [16]. These cells secrete pro-inflammatory and pro-fibrotic factors that activate other mesenchymal cells of the interstitium and promote their differentiation into myofibroblasts. EMT is sustained by multiple intracellular pathways, including Wnt/β-catenin signaling, considered one of the main players in this process. After an acute insult, a temporary activation of this pathway is considered of fundamental importance in renal repair [17]. If the injury persists, Wnt/β-catenin signaling favors transformation of TECs into a mesenchymal secretory phenotype. Moreover, it stimulates differentiation of interstitial fibroblasts into myofibroblasts and activation of pro-fibrotic macrophages [17]. Several experimental studies demonstrated that inhibition of Wnt/β-catenin effectively suppresses EMT and reduces interstitial fibrosis [18], highlighting its major role in EMT.

### 2.2. Macrophages

Other cells involved in renal fibrosis are represented by resident macrophages. These cells are fundamental players in the homeostasis of renal parenchyma, mediating both repair and fibrogenesis after an acute insult [19]. Two different subsets of macrophages exist: M1 macrophages, having a pro-inflammatory activity, and M2 macrophages, mediating anti-inflammatory and tissue-repairing pathways. During acute kidney injury, M1 macrophages release pro-inflammatory chemokines and cytokines, including interleukin (IL)-1β, IL-6, and TNF, promoting an acute inflammatory response. M1 cells are then replaced by M2 macrophages during the subsequent phase of renal repair. In chronic conditions, M2 subset shifts to a pro-fibrotic phenotype mediating recruitment and activation of myofibroblasts, with consequent stimulation of fibrogenesis [19,20]. Persistency of M2 cells has been associated with renal interstitial fibrosis, tubular injury, and collagen deposition [21]. In animal models, depletion of M2 macrophages has been shown to reduce collagen deposition, glomerulosclerosis, and interstitial fibrosis [19,21,22].

Notably, it has been recently demonstrated that macrophages resident in the kidneys constitutively express on their surface the V-domain Ig suppressor of T cell activation (VISTA) [23] and that this molecule seems to play a relevant role in preventing renal fibrosis after an acute glomerular injury [24]. VISTA is an immune checkpoint regulator that can be expressed by myeloid as well as lymphoid cells under various stimuli, and it represents a potent inhibitor of T cells in different conditions, cancer, and autoimmune diseases [25]. VISTA may become expressed by immune cells surrounding or infiltrating tumors and also by neoplastic cells, and its expression is capable of blocking the anti-tumor immune response [25]. On the other hand, VISTA is known to play a significant role in autoimmune diseases, limiting T cells differentiation and activity. In several experimental models, a deficit in its expression has been shown to increase effector response of T cells, eliciting development or exacerbation of autoimmune diseases in susceptible subjects [26,27].

Interestingly, it has been recently demonstrated that VISTA is constitutively expressed, even in normal conditions, on macrophages resident in the kidneys, and at higher levels compared to macrophages in other tissues, while its expression on other immune cells in the kidney is minimal or absent [23]. VISTA+ macrophages inhibit activation and proliferation of T cells and contribute to renal repair in ischemia-reperfusion injury [23]. Moreover, VISTA+ macrophages seem to be involved not only in kidney repair process, but also in prevention of fibrosis development. In a murine model of immune-complexes (ICs) acute glomerular injury, mice deficient for VISTA experienced more severe glomerular and tubular injury and developed severe tubulointerstitial fibrosis. Specifically, VISTA-deficient animals showed increased number of CD4+ and CD8+ T cells infiltrating renal parenchyma, and higher production of interferon (IFN)-γ by T cells, with consequent increased production of IL-9 by parenchymal cells, and progression to severe interstitial fibrosis. On the contrary, mice with preserved expression of VISTA developed less extended fibrosis. Therefore, VISTA+ macrophages seem to have a key role in renal repair and prevention of fibrosis development after acute kidney injury.

### 2.3. Transforming Growth Factor-β

**Figure 1:**
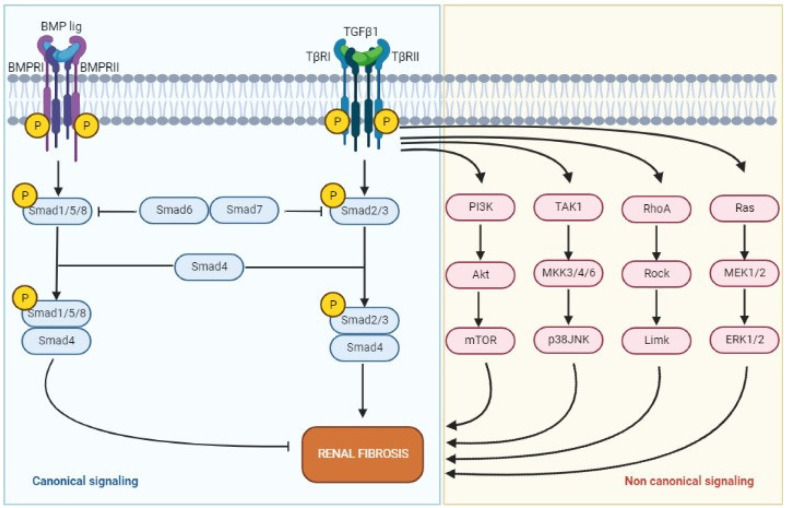
Representation of the TGF-β/Smad signaling. TGF-β1 binds to its receptors and induces a cascade of signal transductions via both canonical (blue) and noncanonical (red) signaling. When TGF-β1 binds to its receptors, it triggers the phosphorylation and activation of Smad2/3, which translocate to the nucleus, inducing the transcription of TGF-β1-responsive genes and a pro-fibrotic response. This process can be blocked by Smad7, who inhibits Smad2/3. On the other hand, when BMP7 binds to its receptors triggers the phosphorylation on Smad1/5/8, which induce an anti-fibrotic response. In addition, TGF-β also activates the noncanonical signaling pathways able to influence renal inflammation and fibrosis. In this case, renal fibrosis is caused by the activation of pathways such as MAP kinase, PI3K/Akt, and Rho kinase, and is triggered by mediators such as angiotensin II and advanced glycation end products (AGEs) [Created with Biorender (https://biorender.com), accessed on 7 November 2022].

Considering known pathways of fibrogenesis, TGF-β stands as one of the most important players of this detrimental process [28]. Produced by macrophages and mesangial cells, under pro-inflammatory stimuli, TGF-β activates intracellular pathways, mainly Smad-mediated signaling, that induce transcription factors for production of ECM components, such as collagen and glycoproteins, increasing ECM deposition, and it inhibits the activity of metalloproteinases, reducing ECM degradation [28]. Moreover, TGF-β/Smad elicits transformation of epithelial cells into myofibroblasts, favoring EMT [29]. TGF-β has been found to sustain renal disease progression to fibrosis also by driving tubular atrophy, podocyte depletion, and loss of capillary endothelial cells [30], with consequent tubular hypoxia and hypoxic-mediated fibrogenesis. An upregulation of expression of TGF-β have been demonstrated in glomerular tufts and tubular compartment of fibrotic kidneys in different conditions [31]. TGF-β signaling is under extensive investigation in renal fibrosis and in other conditions and represents one of the possible future target therapies against fibrosis. Figure 1 resumes the TGF-β/Smad signaling.

### 2.4. Hypoxia

In kidneys, as in other organs, hypoxia plays a major role in triggering and perpetuating pro-fibrotic mechanisms. It should be noted that several niches in renal parenchyma in normal conditions cope with very low oxygen levels, as low as 10 mmHg in the tubular compartment of renal medulla [32]. When oxygen levels further reduce for any reason, an imbalance between cellular demand and supply, i.e., hypoxia, occurs, triggering pro-inflammatory and pro-fibrotic stimuli through different pathways [33]. TECs are particularly susceptible to hypoxia. When glomerular tufts are injured, blood supply to peritubular capillaries of their associated tubular compartments is compromised, leading to local hypoxia. TECs exposed to hypoxia produce higher levels of hypoxia-inducible factor 1 (HIF 1), which in turn promotes EMT and fibrogenesis through several pathways [34,35]. HIF-1 upregulates production of pro-fibrotic cytokines and factors, such as TGF-β, that promote fibrogenesis. It activates macrophages, which in turn mediate other pro-fibrotic stimuli, and release inflammatory cytokines, including IL-1β, which promotes EMT and fibronectin deposition [36]. Moreover, HIF-1 favors ECM expansion through induction of nuclear factor κappa B (NF-κB) signaling, which decreases the activity of metalloproteinases, thus inhibiting ECM degradation [37].

HIF is also able to induce the production of other factors that contribute to fibrosis, including the transcription factor Twist and B lymphoma Mo-MLV insertion region homolog 1 (Bmi), which lead to downregulation of E-cadherin expression and upregulation of fibronectin and vimentin in TECs, promoting EMT [38].

### 2.5. Towards CKD

Regardless the nature of the insult, a progressive loss of nephrons leads to impairment of kidney function, which results in CKD and eventually ESRD. Once CKD is established, it perpetuates progressive loss of functioning nephrons in a maladaptive mechanism of hyperfiltration of the remaining units trying to compensate lost nephrons [39]. Hyperfiltration entails an increased shear stress in the glomerular tuft, with consequent injury and detachment of podocytes, which constitute a fundamental part of the glomerular filtration barrier. Detachment of podocytes leads to glomerular focal and then global scarring, with final loss of function, in a vicious cycle that further increases the filtration load for the remaining functioning nephrons and progressive fibrosis [40]. Hyperfiltration is a complex phenomenon sustained by different players, including angiotensin II, a fundamental component of the renin–angiotensin–aldosterone system (RAAS) [7]. Angiotensin II promotes vasoconstriction of efferent arterioles of glomerular tufts, increasing glomerular hydrostatic pressure and promoting hyperfiltration. At the same time, angiotensin II is a pro-inflammatory and pro-fibrotic mediator. It elicits, in fact, intracellular signals that promote deposition of ECM, including αSMA and fibrogenic connective tissue growth factor signaling [41]. ECM overproduction and EMT result in fibrosis of the tubulointerstitial compartment, with reduction of capillary density, worsening of hypoxia, and sustained fibrogenesis [7].

Interestingly, it has been recently hypothesized that fibrosis originating from glomerular injury and consequent atrophy of related tubules differs from fibrosis originating from initial injury to the tubules, as in obstructive uropathy or tubular toxicity. In a murine model comparing injuries in different renal compartments (primary glomerular injury, obstructive uropathy, and direct tubular damage) Tampe et al. [42] found different histological patterns of fibrosis. Glomerular injury and direct tubular toxicity were associated with focal IF/TA corresponding to the involved tubular complexes. On the other hand, obstructive uropathy was associated with diffuse fibrosis of the interstitium surrounding intact tubules. Moreover, ultrastructural analysis and transcriptome analysis of fibrotic compartments in these models revealed a different composition and organization of ECM and distinct collagen signatures in focal and diffuse IF/TA, suggesting different pathogenic pathways sustaining fibrogenesis. Authors hypothesized that, while focal IF/TA represents a permanent scarring of parenchyma following the loss of functioning nephrons, either due to glomerular or direct tubular injury, diffuse fibrosis involving primarily the interstitium may be a reversible process [42]. These observations in kidneys are similar to other findings in studies on cardiac fibrosis, where permanent focal fibrosis following cardiomyocyte necrosis and reversible diffuse fibrosis surrounding normal cardiomyocytes have been described [43]. This field surely deserves further investigation.

## 3. Fibrosis in Lupus Nephritis

SLE is a prototypical autoimmune disease with a complex and multifactorial pathogenesis characterized by predisposing genetics, altered self-tolerance with production of autoantibodies, antinuclear antibodies (ANA), including anti-dsDNA antibodies, immune complexes (ICs), mediated injury to different organs and tissues, and deficient phagocytosis of ICs and cellular debris [44]. Approximately 40% of adults and 80% of children suffering from SLE have a renal involvement of the disease, defined as lupus nephritis (LN) [9]. Kidney involvement usually presents early in the disease course and often results to be fundamental in the diagnosis and prognosis of SLE. Pathogenesis of LN entails deposition of nucleic acid-containing material and ICs in the glomeruli, with activation of the complement system and recruitment of pro-inflammatory cells, consequently causing glomerular injury and tubulointerstitial damage. Subendothelial deposition of ICs prompts the recruitment of pro-inflammatory cells, leading to what is defined as the proliferative phase of the disease, with increased glomerular cellularity, including mesangial proliferation, leukocyte recruitment, and endothelial proliferation. On the other hand, when ICs deposit in subepithelial space, they can cause podocyte injury with foot process effacement and expansion of glomerular basement membrane (GBM), with consequent disruption of glomerular filtration barrier and nephrotic proteinuria [10,45].

Fibrosis in LN, as in other kidney diseases, represents a terminal pathway of sustained immune-mediated injury and it has been recognized as a determinant factor in defining response to therapy and renal prognosis [46,47,48]. A better understanding of cellular and molecular pathways involved in fibrosis in LN could enable the identification of individuals at higher risk of progression to CKD and ESRD, and the development of new therapeutic strategies for patients with SLE.

### 3.1. Detrimental Role of Autoantibodies

Considering the ICs-mediated pathogenesis of LN, the potential role of anti-double stranded DNA (anti-dsDNA) antibodies in fibrogenesis has been investigated. These autoantibodies are able to bind cross-reactive antigens expressed on surfaces of resident renal cells. It has been demonstrated that they can bind, for example, annexin II and α-actin on mesangial cells, A and D SnRNP polypeptides on TECs, and membrane proteins on endothelial cells [49,50,51]. Binding of anti-dsDNA elicits intracellular signaling that promotes cell proliferation and activation of profibrotic pathways, including production of fibronectin and collagen through activation of TGF-β, MAPK, and PKC-mediated pathways [49]. Moreover, anti-dsDNA binding to TECs stimulate EMT. Notably, in kidney biopsies, 70% of patients with LN have ICs deposits not only in glomerular tufts, but also along tubular basement membranes, suggesting the key role of immune-mediated inflammation affecting also tubular compartment and leading to IF/TA [52].

### 3.2. Tubular Epithelial Cells

As for renal fibrosis in other kidney diseases, TECs secrete cytokines involved in fibrogenesis, including TGF-β, IL-6, IL-8, and connective tissue growth factor, recruiting more inflammatory cells and enhancing secretion of ECM components, in a vicious cycle that evolves into fibrosis [53].

Single-cell RNA sequencing (sc-RNA seq) has been recently used to better define pathways and genes involved in LN fibrosis [54]. With this technique, transcriptomic data and gene expression of specific cell lines can be analyzed. Sc-RNA seq was used to analyze samples of renal biopsies from patients with LN and healthy controls, analyzing differences in genes and transcriptomic expressions in TECs, which constitute the most represented cell type in kidney biopsy [55]. The study demonstrated that TECs from LN patients highly expressed upregulation of type 1 IFN-response genes, compared to healthy controls. Degree of expression of IFN-response genes in TECs was used to develop an IFN-response score to be applied to each LN patient. Notably, individuals who did not respond to therapy showed significantly higher IFN-response scores compared to partial or complete responders, and they had also an upregulation of genes encoding for ECM proteins, such as TIMP and SERPIG, known to be associated with renal fibrosis [56,57]. Some of the non-responder patients with upregulated pro-fibrotic genes did not show histological signs of IF/TA at kidney biopsy. Moreover, those who had higher IFN-response scores at the time of biopsy were less likely to respond to therapy at 6 months. These evidences highlighted TECs as central players of fibrosis in LN and highlighted how sc-RNA seq may predict future development of fibrosis before it becomes clinically evident, and it may predict resistance to current therapy, prompting a different, more aggressive approach in such patients [55].

### 3.3. Type 1 Interferon

Type 1 IFN regulates numerous pro-inflammatory pathways in leukocytes and in other cells, being a key player of the inflammatory cascade. Its production is usually stimulated by DNA and RNA debris, which can be easily found in SLE, where cellular debris of apoptotic cells are not effectively cleared. In LN, type 1 IFN has been shown to participate in maintenance of a pro-inflammatory and pro-fibrotic environment [58], favoring recruitment of leukocytes, inhibiting anti-inflammatory action of M2 macrophages [59], and inducing expression of B cell activating factor (BAFF) [60], thus favoring autoantibody production. Through numerous pro-inflammatory pathways, including TGF-β signaling, type 1 IFN stimulates pericytes proliferation and differentiation to myofibroblasts, and it mediates injury to renal resident cells, including podocytes, endothelial cells, and mesangial cells, contributing to progression of kidney injury and consequent fibrosis in LN [58].

### 3.4. TGF-β

Another key player in LN fibrosis, as in renal fibrosis, is TGF-β. As previously discussed, TGF-β activates Smad signaling, stimulating ECM deposition, EMT, and other pro-fibrotic pathways that lead to renal fibrosis [28]. In LN, TGF-β/Smad signaling has been found to promote proliferation of mesangial cells (MCs), which represent fundamental constituents of glomerular architecture and main source of mesangial ECM [61]. When stimulated, MCs proliferate and increase deposition of ECM, leading to glomerular remodeling and scarring. Moreover, TGF-β mediates injury to podocytes and TECs, and loss of peritubular capillaries, stimulating hypoxia-driven fibrotic pathways [35]. Production of TGF-β in LN can be induced by anti-dsDNA antibodies binding to TECs and MCs, and by type 1 IFN [49,58].

Interestingly, exosome sequencing on urine samples of patients with LN showed that detected miRNAs were precisely linked to TGF-β/Smad pathways [62]. With this technique, three miRNA sequences, miR-21, miR-150 and miR-29, were found to be specifically expressed in samples of LN patients and can represent easily detectable biomarkers of fibrosis in LN. These miRNAs, in fact, were proved to correlate with degrees of chronicity index at kidney biopsy, and to be able to predict progression to ESRD [62].

### 3.5. Complement System

Complement is known to be a fundamental mediator of kidney injury also in LN [63]. Activation of complement pathways leads to formation of membrane attack complex (MAC), which mediates kidney injury through different mechanisms, including cytokine release and cell-lysis, on resident renal cells. Its role in the development of interstitial fibrosis in LN has been recently suggested. When MAC forms on TECs, it favors tubular interstitial injury and consequent IF/TA [64]. In a pilot study on kidney biopsies of LN patients, MAC was detected along tubular basement membranes in 23% of patients, independently from deposition in glomerular tufts. Patients with MAC deposition on TECs had higher proteinuria, and a higher degree of IF/TA and a chronicity index at kidney biopsy [64]. These findings suggest that complement terminal pathway participates in the development of interstitial injury and fibrosis. More studies are needed to further elucidate its role in promoting fibrogenesis and as new therapeutic target against progression of IF/TA and ESRD.

### 3.6. Dickkopf-Related Protein 3

A potential new marker of fibrosis and CKD progression in LN is represented by Dickkopf-related protein 3 (DKK3). DKK3 is a regulatory protein involved in different cellular pathways, including cell cycle and differentiation, and it is known to interact with the Wnt-β catenin pathway [65,66], implicated in development and progression of CKD after sustained renal injury, as we mentioned. In a prospective study on 132 SLE patients, 57 with LN, significantly higher serum levels of DKK3 were detected in patients with renal involvement compared to SLE patients without LN and to healthy controls [67]. Higher levels of DKK3 were able to identify individuals with a higher degree of renal fibrosis and chronicity index at kidney biopsy. Moreover, levels of DKK3 correlated with worsening kidney function and progression of CKD over a 3-year follow up [67]. DKK3 may represent a new and easy to use marker for diagnosis of renal involvement in SLE patients and for prognosis of LN, and further studies are needed to confirm its reliability.

**Figure 2:**
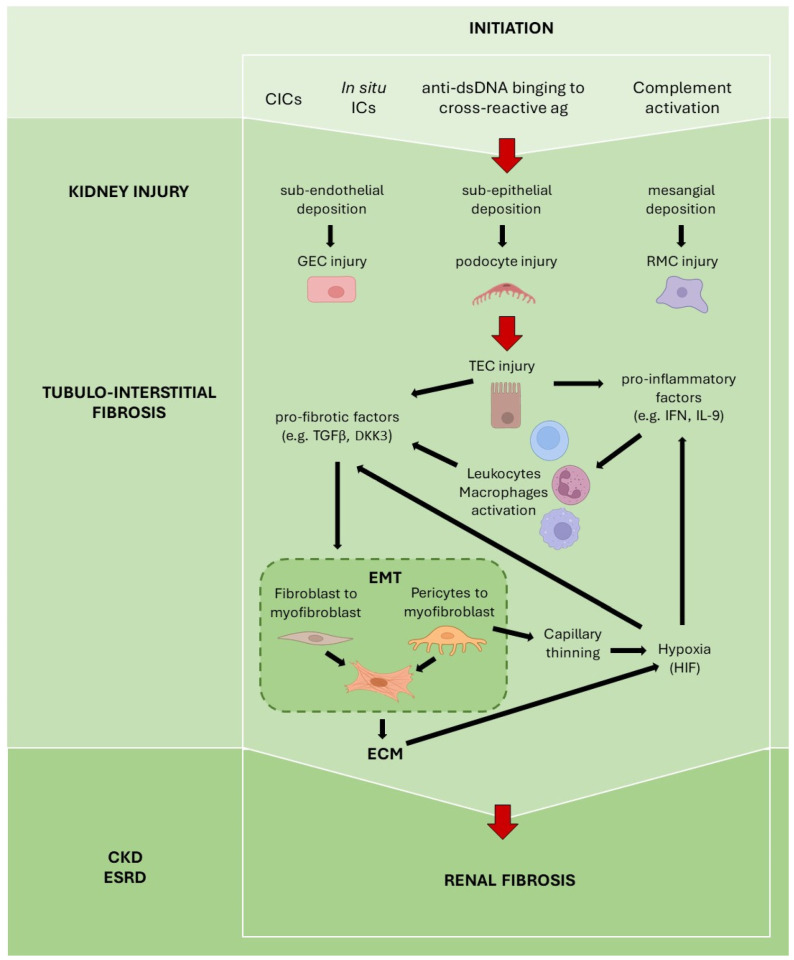
Main mechanisms involved in the pathogenesis of renal fibrosis in LN. Abbreviations: CIC—circulating immune complex; CKD—chronic kidney disease; ECM—extracellular matrix; EMT—epithelial-to-mesenchymal transition; ESRD—end-stage renal disease; GEC—glomerular endothelial cell; HIF—hypoxia-inducing factor; IC—immune complex; RMC—renal mesangial cell; TEC—renal tubular epithelial cell [Created with Biorender (https://biorender.com) accessed on 7 November 2022].

Figure 2 resumes the main mechanisms involved in renal fibrosis in LN.

## 4. Histological Classification—IF/TA as a Prognostic Factor in LN

Kidney biopsy still represents the gold standard for assessing diagnosis and prognosis of LN [68]. The histological scoring system is based on the International Society of Nephrology/Renal Pathology Society (ISN/RPS) classification [11]. Six histological categories can be distinguished, namely: class I—minimal mesangial LN; class II—mesangial proliferative LN; class III—focal LN; class IV—diffuse LN; class V interstitium—membranous LN; and class VI—advanced sclerosing LN.

The goal of the histological ISN/RPS classification was not only to provide LN diagnosis, but also to highlight prognosis and, possibly, response to therapy. Until 2018, the ISN/RPS classification had been mainly focused on glomerular lesions, without much consideration for tubulointerstitial and vascular injuries. However, during years, this ‘glomerulocentric’ system has been challenged by many authors and international experts. Many practical issues have been raised on the short-term prognostic value of this classification, on inconsistencies with response to treatment, and on lack of acknowledgement for the role tubulointerstitial and vascular parameters in predicting renal outcomes. For example, in an investigation by Yu et al. on 313 LN biopsies, interstitial infiltration, tubular atrophy, and interstitial fibrosis were found to be an independent risk factors for renal prognosis [69]. Hsieh et al. showed that risk of ESRD was predicted by tubulointerstitial inflammation and fibrosis, and not by glomerular injury, in a cohort of 68 LN patients [70]. In another report on 105 subjects with LN, Rijnink et al. observed that fibrous crescent and IF/TA ≥ 25% were predictive of progression to ESRD, as well as estimated glomerular filtration rate (eGFR) at baseline and non-white race, irrespectively of LN class [46]. On these and several other observations, in 2018 a revision of ISN/RPS classification was released [11]. Among the main changes introduced, the ISN/RPS working group endorsed the adoption of activity and chronicity indexes used by the National Institutes of Health’s (NIH) classification of LN. The working group adopted a modified version of the original NIH indexes based on previous work by Austin et al. [71], which had suggested a semiquantitative assessment of different parameters to score active and chronic lesions. Each considered parameter can be scored as 0–3, with the overall activity index having 0–24 points and the overall chronicity index having 0–12 points. The ISN/RPS working group acknowledged the need for a scoring system of LN that included also the evaluation of tubulointerstitial injury. Specifically, the adopted activity index, constituted by six parameters, includes also interstitial inflammation, while the chronicity index, with four parameters, takes into consideration glomerulosclerosis, fibrous crescents, tubular atrophy, and interstitial fibrosis. Moreover, the ISN/RPS working group underlined that histological evaluations should indicate if interstitial inflammation, i.e., infiltrates of leukocytes in the interstitium, occurs in the presence or absence of interstitial fibrosis, as this may have a different meaning in terms of response to therapy and renal functional prognosis [11].

Following the new revised classification of LN, several studies have been performed, focusing on the prognostic value of tubulointerstitial injury and fibrosis in predicting renal outcome and progression to ESRD. Wilson et al. retrospectively examined the effect of interstitial inflammation and IF/TA on renal survival in 218 LN patients [72], demonstrating the independent prognostic value of the two parameters. The group divided patients into three risk categories based on percentages of tubular injury detected in histological samples. The results showed that patients with the highest percentage of tubular inflammation and/or IF/TA had the highest risk of renal failure and mortality at a long-term follow up. Similarly, in a study on 131 LN patients, Broder et al. found that moderate to severe IF/TA at kidney biopsy was associated with a significantly higher risk of progression to ESRD, especially in individuals with preserved eGFR (≥60 mL/min/1.73 m^2^) at baseline, with an adjusted hazard ratio (HR) of 6.2 [73]. Interestingly, no correlation was found between interstitial inflammation and ESRD in this study. Another work from Leatherwood et al. on 202 LN biopsies confirmed similar findings, as moderate to severe IF/TA was associated with development of ESRD (HR 5.18) and death (HR 4.19), irrespectively of LN histological class [74]. In another experience on 166 LN patients, Gomes et al. found that almost 70% of kidney biopsies showed tubulointerstitial lesions, either inflammation or IF/TA. Renal survival in this study was inferior in individuals with moderate to severe IF/TA [75]. Moreover, conversely to Broder et al.’s findings, grade of tubulointerstitial inflammation was independently linked to ESRD, after adjustment for degree of IF/TA, hypertension and diabetes [75].

Finally, in a recently published work, Moroni et al. clearly demonstrated the association between chronicity index and all its components, taken individually, and CKD/ESRD in a cohort of 203 LN patients on a long-term follow up of 14 years [76]. Notably, interstitial inflammation was noted to be the only parameter of the activity index associated with the development of CKD. However, the association with renal impairment depended on the presence of IF/TA, while it was lost in patients with preserved tubulointerstitial spaces. As the authors suggest, similarly to what has been observed in kidney transplantation [77], interstitial inflammation without IF/TA represents active lesions susceptible to prompt therapy, while infiltrates in a fibrotic interstitium usually are not responsive to therapy and correlate with renal failure [76]. These data reinforce the abovementioned suggestions made by the ISN/RPS working group to specify if interstitial inflammation occurs in the presence or absence of interstitial fibrosis in kidney biopsies.

All these and other evidences underline the importance of evaluating tubulointerstitial injury, in addition to clinical parameters, in order to define the prognosis of LN in terms of renal survival. Many aspects have to be more clearly elucidated, including the role of interstitial inflammation with and without interstitial fibrosis, and if tubular atrophy and interstitial fibrosis can be considered as a unique entity (IF/TA) or if they deserve to be evaluated separately. Given the strong association between moderate to severe IF/TA and ESRD, being able to target fibrogenesis and to stop progression of fibrotic changes in the parenchyma may represent a fundamental part of future therapies in order to preserve kidney function, beyond the underling initial insult, in LN setting. We will briefly address this point in the final paragraph of this review.

## 5. Future Targeted Therapies in Renal Fibrosis

As fibrosis represents a significant determinant in renal functional outcomes, its targeting with specific therapies may represent an interesting option for patients suffering from renal diseases, including LN, and affected by CKD. The complexity of pathways involved in the genesis and progression of renal fibrosis, combined with the paucity of reliable and quantifiable non-invasive biomarkers, makes the identification of treatment strategies in this field a real challenge. At present, there are no agents specifically approved for prevention and treatment of renal fibrosis in humans. Drugs under investigation with possible effects on renal fibrosis in different kidney diseases are briefly summarized in Table 1.

### 5.1. Anti-Fibrotic Drugs

Anti-fibrotic drugs have been approved for the treatment of fibrosis in other organs, but not the kidney. Specifically, Pirfenidone, a TGF-β inhibitor and scavenger of reactive oxygen species, and Nintedanib, a tyrosine kinase inhibitor, were approved by the FDA for treatment of idiopathic pulmonary fibrosis [78,79,80]. Limited data exist on the employment of these drugs in kidney disease. Some beneficial effects against progression of CKD have been detected with the use of Pirfenidone in patients with focal segmental glomerulosclerosis (FSGS) in an observational uncontrolled study, in the absence, however, of any effect on proteinuria or blood pressure [81]. In this study, a second kidney biopsy was not performed to assess the effect of Pirfenidone on progression of kidney disease and fibrosis.

Nintedanib is a potent intracellular inhibitor of tyrosine-kinases implicated in several critical pathways involved in the pathogenesis of pulmonary fibrosis, including cell proliferation, differentiation, apoptosis, fibroblast activation, and ECM production [82,83]. It is currently approved for idiopathic pulmonary fibrosis and interstitial lung diseases (ILDs), including systemic sclerosis ILD and chronic fibrosing ILDs with a progressive phenotype [80,84,85]. Since Nintedanib inhibits fundamental processes of lung fibrosis in vitro, in animal models and in patients with ILDs, it could hopefully be proposed as a therapeutic tool against renal fibrosis. However, its use in kidney diseases has been limited by concerns of its possible association with renal thrombotic microangiopathy, which deserves further investigation [86,87]. Studies of Nintedanib, alone or in combination with Gefitinib, an epidermal growth factor inhibitor, have shown beneficial effects on kidney fibrosis in experimental murine models of obstructive uropathy [88]. At present, there are no ongoing trials of these agents with an indication to specifically treat renal fibrosis.

### 5.2. Chimeric Antigen Receptor T Cells

Another new and revolutionary anti-fibrotic approach is represented by the use of chimeric antigen receptor (CAR) T cells. This innovative therapy employs T lymphocytes engineered to target and kill specific cells. Originally developed to target neoplastic cells, CAR-T lymphocytes have been recently tested against fibrosis in the myocardium and the liver [89,90,91]. In murine models, T lymphocytes were built to target fibroblast activation peptide (FAP) on surface of activated cardiac fibroblasts [89,90] or urokinase-type plasminogen activator receptor on hepatic myofibroblasts [91]. Destruction of targeted fibroblasts by CAR-T cells resulted in the reduction of fibrosis and improved organ function. Interestingly, Rurik et al. recently demonstrated that CAR-T cells may be easily engineered in vivo with the use of lipid nanoparticles directed specifically to T cells and containing mRNA sequences encoding for a CAR against the desired target, FAP in case of cardiac fibrosis [89]. CAR-T cells in fibrosis of course have to be implemented and tested in different organs, as a specific surface molecule for each tissue needs to be defined to minimize the effect on other tissues. Moreover, it is not known what is the extent of fibrosis against which this therapy continues to be effective or, on the contrary, loose efficacy. However, CAR-T cells represent a very promising tool to target fibrogenesis.

### 5.3. Other Agents Targeting Fibrosis in Kidney Diseases and LN

Numerous other anti-fibrotic target therapies are currently under investigation in renal fibrosis and in LN fibrosis.

Fresolimumab, a monoclonal antibody against TGF-β, underwent a phase 2 trial in patients with FSGS [92]. The drug failed to show any beneficial effect on proteinuria, but did seem to stabilize GFR, limiting the progression of renal fibrosis. However, the trial was underpowered to evaluate effects on GFR decline and no serial kidney biopsies were performed to assess changes in renal histology in terms of disease progression or fibrosis.

Bardoxolone methyl, an inhibitor of NF-κB transcription factor, studied in Alport syndrome [93], seemed to slow the progression of CKD in these patients and it is currently under investigation in a phase 3 trial on its efficacy and safety in patients with autosomal dominant polycystic kidney disease (FALCON trial NCT03918447) and in a phase 3 trial on long-term safety in patients with CKD (EAGLE trial NCT03749447).

Many different studies, in animal or in vitro models, tested the efficacy of inhibiting specific pathways or molecules known to be key players in renal fibrosis. These include several molecules involved in the abovementioned Wnt/β-catenin signaling, main player of EMT, other targets of the EMT process [18], and TGF-β signaling [94]. Although promising future therapies, they still remain at pre-clinical stages and need further investigation.

Considering LN, a variety of old and new drugs have been tested against fibrosis in animal models of this condition. Among old drugs, Mycophenolate Mofetil (MMF) deserves particular attention, already representing one of the standard therapies for patients affected by LN [95,96,97]. MMF limits lymphocyte proliferation through inhibition of inosine monophosphate dehydrogenase, an essential enzyme in de novo synthesis of purines. Moreover, it inhibits proliferation of MCs [98], TECs [99], and fibroblasts [100] and it reduces fibronectin synthesis [101]. In a murine model of LN, animals treated with MMF showed reduced mesangial proliferation, ECM expansion, and tubular atrophy, and had markedly reduced expression of TGF-β in both glomerular tufts and tubular compartments [102]. In the same study, human mesangial cells stimulated by anti-dsDNA to produce TGF-β showed a reduced secretion of this cytokine, when incubated with MMF [102]. In other two sequential studies on mice with LN, MMF and the mTOR inhibitor Rapamycin have been proven to be able to reduce fibrosis or delay its progression through a direct effect on mesangial cells [103,104]. Mice treated with either MMF or Rapamycin alone or both drugs in association showed decreased histological signs of fibrosis, with inferior mesangial proliferation and tubular atrophy, and had reduced expression of TGF-β1, α-SMA and fibronectin compared to untreated controls [103,104]. With these results, further research on the effects of MMF as anti-fibrotic agent in patients with LN would be of great interest.

Considering new drugs, other agents were proven to be effective against fibrosis in murine models of LN. For example, Iguratimod, an antirheumatic drug approved for rheumatoid arthritis in Japan and China, was found to effectively reduce renal interstitial fibrosis and tubular atrophy inhibiting collagen fibers deposition and blocking EMT through interference with TGF-β/Smad signaling [105]. Moreover, it reduced ICs deposition along tubular basement membrane and infiltration of inflammatory cells in the interstitium [105].

Finally, several monoclonal antibodies currently tested to treat patients with active LN may also have potential secondary effects against renal fibrosis. Drugs under investigation in ongoing trials include anti–CD20 Obinutuzumab (REGENCY trial, NCT04221477), anti–CD38 Daratumumab (NCT04868838), the type 1 IFN antagonist Anifrolumab (TULIP-LN trial, NCT02547922), anti IL-23 Guselkumab (ORCHID-LN trial, NCT04376827), anti IL-17A Secukinumab (SELUNE trial, NCT04181762). It has not yet been investigated if these new therapies have also an impact on fibrogenetic mechanisms and this surely represents an unexplored and promising field.

## 6. Conclusions

In conclusion, fibrosis represents the final common pathway of altered healing from repeated injuries and chronic inflammation. Mechanisms of fibrogenesis in different organs, including the kidneys, and in different conditions such as LN, have raised more and more attention in the last years, with a constantly growing knowledge in this field. The clinical burden of fibrosis on renal prognosis and response to therapy in LN have been recognized in the last years. Accordingly, the histological ISN/RPS classifications have been updated, with the introduction of a chronicity index, scoring glomerulosclerosis, fibrous crescents, tubular atrophy, and interstitial fibrosis. This index has been subsequently validated in different clinical studies and it has been proved to correlate with renal outcome and progression to ESRD. While the key role of fibrosis in clinical outcomes is clear, at the present time, no specific treatment exists for it in kidney diseases, including LN. Being able to stop fibrosis or at least slow its progression, with its consequent deleterious impact on organ function, will represent a turning point in the treatment of many invalidating conditions, such as CKD, or threatening diseases, such as SLE. This new and intriguing field of research surely deserves further investigation.

## Figures and Tables

**Table 1:** 2022 status of drugs in clinical development for kidney fibrosis (status of each study is reported as recorded on ClinicalTrials.gov, accessed on 20 October 2022).

Agents	Clinical Development
Pirfenidone	- Trial of Pirfenidone to Prevent Progression in Chronic Kidney Disease: RECRUITING - Utility of Prolonged-release Pirfenidone in the Progression of Chronic Kidney Disease: COMPLETED - Pirfenidone: A New Drug to Treat Kidney Disease in Patients With Diabetes: COMPLETED - Effect of Pirfenidone on Glomerular Filtration Rate and Albuminuria in Patients With Diabetic Nephropathy: unknown - Pirfenidone Capsule in Patients With Chronic Kidney Disease G2 and G3a Study on Safety and Pharmacokinetics: RECRUITING - Pirfenidone to Treat Kidney Disease (Focal Segmental Glomerulosclerosis): COMPLETED and HAS RESULTS - Pirfenidone Effect on the Recovery of Renal Function in Septic Acute Kidney Injury: unknown - Permeability Factor in Focal Segmental Glomerulosclerosis: COMPLETED and HAS RESULTS - Utility of Prolonged-release Pirfenidone in the Progression of Chronic Kidney Disease: COMPLETED
Fresolimumab, LY2382770	- A Study of Fresolimumab in Patients With Steroid-Resistant Primary Focal Segmental Glomerulosclerosis (FSGS). COMPLETED - Safety Study of GC1008 in Patients With Focal Segmental Glomerulosclerosis (FSGS) of Single Doses of GC1008 in Patients With Treatment Resistant Idiopathic FSGS. COMPLETED
FG-3019 (pamrevlumab)	- Safety and Pharmacokinetics of FG-3019 in Adolescents and Adults With Focal Segmental Glomerulosclerosis (FSGS): TERMINATED - Phase 1 Study of FG-3019 in Subjects With Type 1 or Type 2 Diabetes Mellitus and Diabetic Nephropathy: COMPLETED - Study of the Safety of FG-3019 in Incipient Nephropathy Due to Type 1 or Type 2 Diabetes Mellitus: COMPLETED - Study of FibroGen (FG)-3019 in Subjects With Type 2 Diabetes Mellitus and Kidney Disease on ACEi and/or ARB Therapy: TERMINATED
GCS-100	- A Phase 2 Extension of Study GCS-100-CS-4003 in Chronic Kidney Disease: WITHDRAWN - A Phase 2 Extension Study of Study GCS-100-CS-4002 in Chronic Kidney Disease: COMPLETED - Safety Study of GCS-100 to Treat Chronic Kidney Disease: COMPLETED - A Phase 2a Study of Weekly Doses of GCS-100 in Patients With Chronic Kidney Disease: COMPLETED - A Phase 2b Study of GCS-100 in Patients With Chronic Kidney Disease Caused by Diabetes: unknown
Fingolimod	- Fingolimod for the Abrogation of Interstitial Fibrosis and Tubular Atrophy Following Kidney Transplantation: Not yet recruiting
Bardoxolone methyl	- A Trial of Bardoxolone Methyl in Patients With CKD at Risk of Rapid Progression (MERLIN trial) COMPLETED - A Phase 2/3 Trial of the Efficacy and Safety of Bardoxolone Methyl in Patients With Alport Syndrome (CARDINAL trial) COMPLETED - A Trial of Bardoxolone Methyl in Patients With ADPKD (FALCON trial). RECRUITING - An Extended Access Program for Bardoxolone Methyl in Patients With CKD (EAGLE trial) RECRUITING - Phase IIa Trial to Determine the Effects of Bardoxolone Methyl on Renal Function in Patients With Diabetic Nephropathy. COMPLETED - Phase II Pharmacodynamic Trial to Determine the Effects of Bardoxolone Methyl on eGFR in Patients With Type 2 Diabetes and Chronic Kidney Disease. COMPLETED

## Data Availability

Not applicable.
